# Supplementary figures and images for: Modeling Approaches Reveal New Regulatory Networks in Aspergillus fumigatus Metabolism
Source: J Fungi (Basel). 2020 Jul 14;6(3):108. doi: 10.3390/jof6030108 (PMC7557846; doi:10.3390/jof6030108)

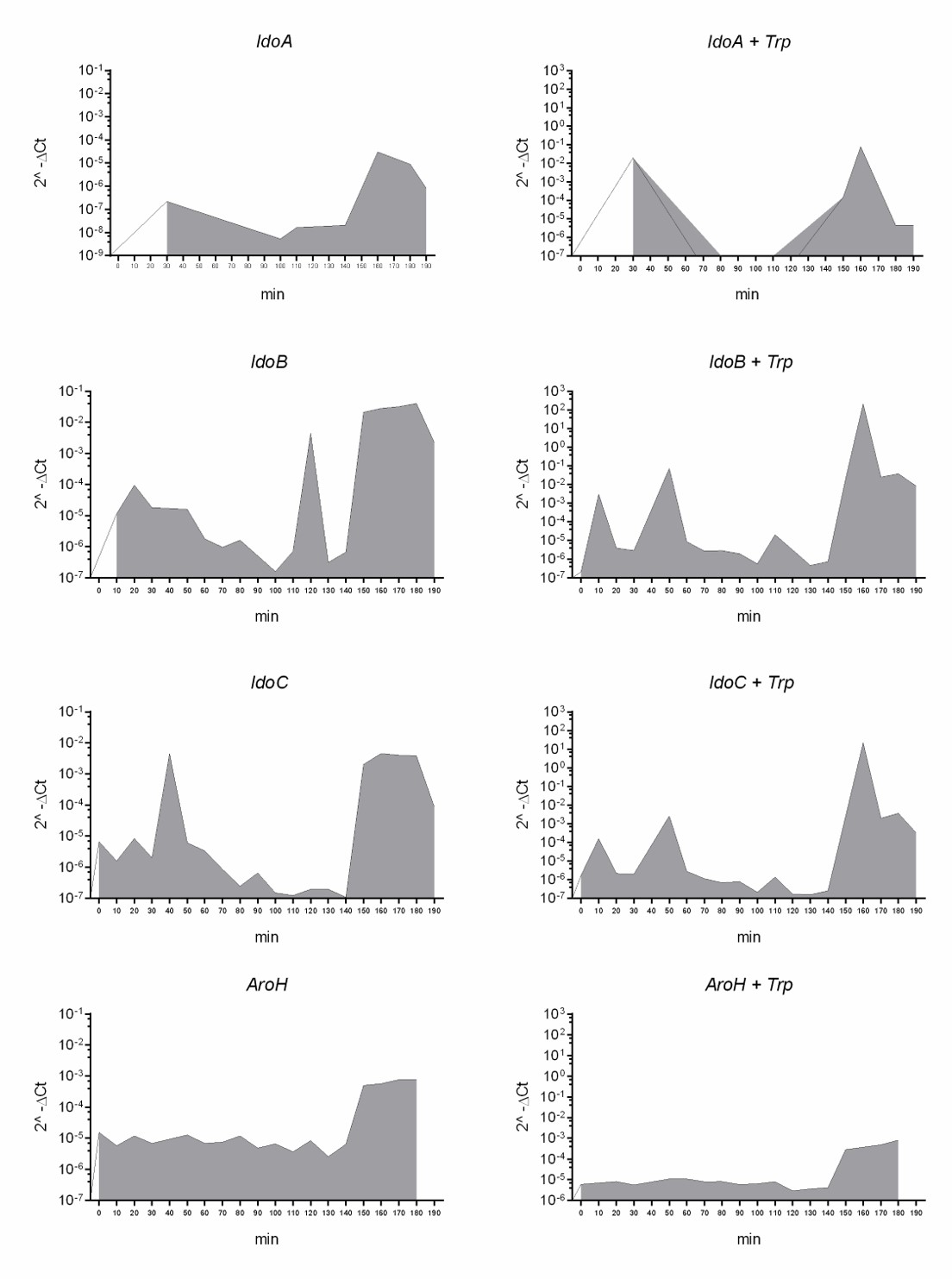

Supplement: Supplementary file 1 [file jof-06-00108-s001.zip › Fig.S1.jpg]

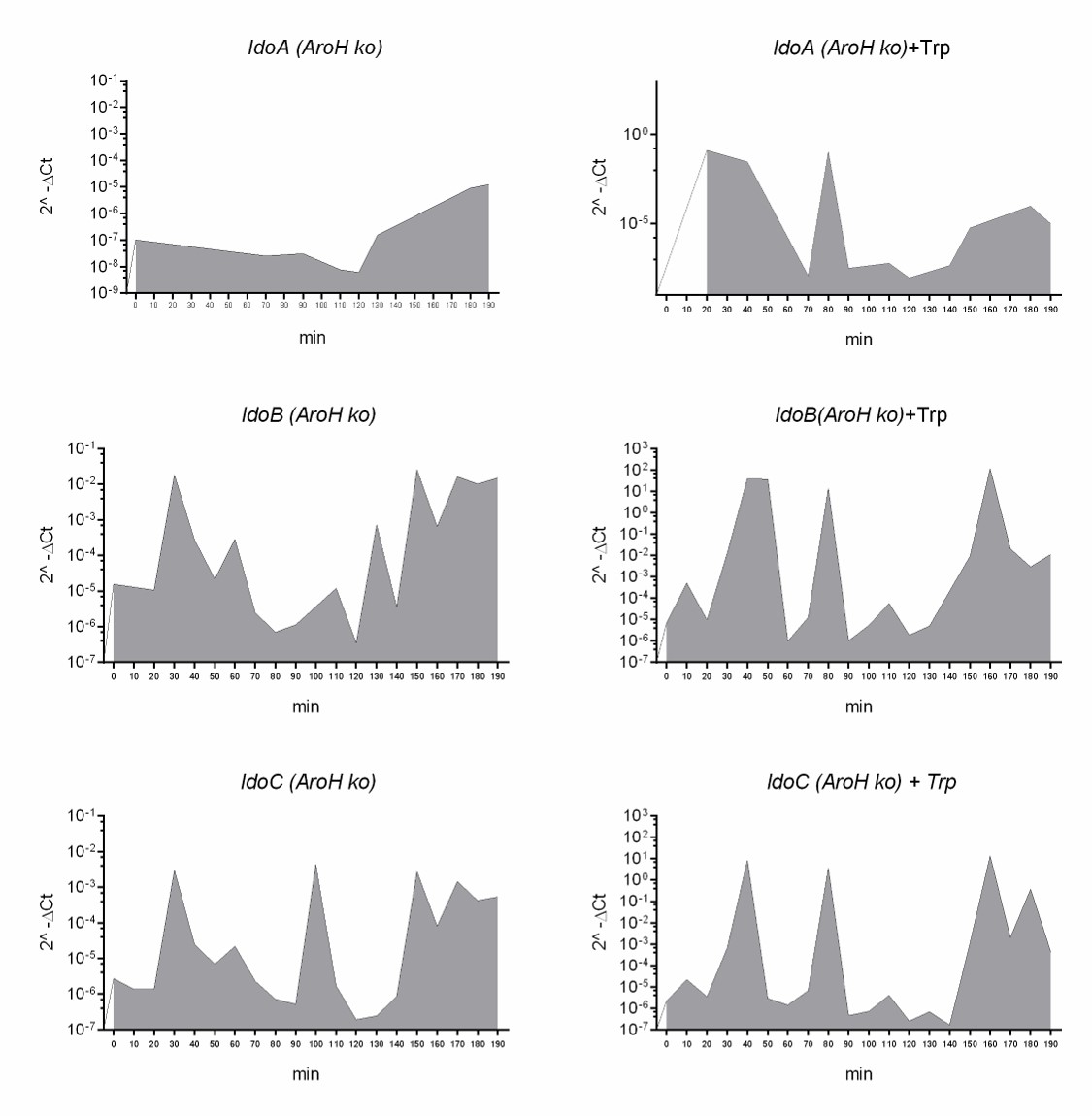

Supplement: Supplementary file 1 [file jof-06-00108-s001.zip › Fig.S2.jpg]
